# Elevated Levels of Activated and Pathogenic Eosinophils Characterize Moderate-Severe House Dust Mite Allergic Rhinitis

**DOI:** 10.1155/2020/8085615

**Published:** 2020-08-13

**Authors:** Yang Chen, Meng Yang, Jie Deng, Kanghua Wang, Jianbo Shi, Yueqi Sun

**Affiliations:** ^1^Otorhinolaryngology Hospital, The First Affiliated Hospital, Sun Yat-sen University, Guangzhou 510080, China; ^2^Guangzhou Key Laboratory of Otorhinolaryngology, Guangzhou 510080, China; ^3^Zhongshan School of Medicine, Sun Yat-sen University, Guangzhou 510080, China; ^4^Department of Otolaryngology, The Seventh Affiliated Hospital, Sun Yat-sen University, Shenzhen 518107, China

## Abstract

Eosinophils play a critical role in the pathogenesis of allergic airway inflammation. However, the relative importance of eosinophil activation and pathogenicity in driving the progression of disease severity of allergic rhinitis (AR) remains to be defined. We aimed to assess the relation of activated and pathogenic eosinophils with disease severity of patients with AR. Peripheral blood and nasal samples were collected from patients with mild (*n* = 10) and moderate-severe (*n* = 21) house dust mite AR and healthy control subjects (*n* = 10) recruited prospectively. Expressions of activation and pathogenic markers on eosinophils in the blood and nose were analyzed by flow cytometry. The eosinophilic cation protein- (ECP-) releasing potential and the pro-Th2 function of blood eosinophils were compared between the mild and moderate-severe patients and healthy controls. Our results showed that the numbers of activated (CD44^+^ and CD69^+^) and pathogenic (CD101^+^CD274^+^) eosinophils in the blood and nose as well as blood eosinophil progenitors were increased in moderate-severe AR compared with the mild patients and healthy controls. In addition, the levels of activated and pathogenic eosinophils in the blood were positively correlated with the total nasal symptom score and serum ECP and eosinophil peroxidase (EPX) levels in patients with AR. Furthermore, the blood eosinophils obtained from the moderate-severe patients exhibited a higher potential of releasing ECP and EPX induced by CCL11 and of promoting Th2 responses than those from the mild patients and healthy controls. In conclusion, patients with moderate-severe AR are characterized by elevated levels of activated and pathogenic eosinophils, which are associated with higher production of ECP, EPX, and IL-4 in the peripheral blood.

## 1. Introduction

Allergic rhinitis is a common allergic disease in both China and Western countries, affecting approximately 10% to 40% of the global population, with over 400 million persons worldwide suffering from symptoms of sneezing, rhinorrhea, nasal obstruction, and aggravation of comorbid asthma [1–5]. Although not life threatening, the disease has significant socioeconomic and quality-of-life impacts [1, 6]. The severity of allergic rhinitis is typically classified into a mild and a moderate-severe form based on symptom severity according to the Allergic Rhinitis and its Impact on Asthma (ARIA) guidelines [1]. Over half of the patients have the moderate-severe form of the disease, leading to significantly impaired normal daily activity [7, 8]. Hence, clinical management of the moderate-severe patients is outstandingly challenging. Pathophysiologically, allergic rhinitis is an IgE-mediated type 2 inflammatory disorder of the nasal mucosa caused by the interaction of airborne allergens characterized by inflammatory infiltrates comprising predominantly eosinophils, mast cells, basophils, and T cells, which release granule proteins, cytokines, and chemokines to trigger the onset of clinical symptoms [9]. However, the mechanism driving the development of the moderate-severe form remains unclear.

It has been well established that eosinophils play an important role in chronic allergic diseases [10, 11]. The number of eosinophils in nasal smear was shown to be highly correlated with the nasal airflow resistance and the spirometric indexes in patients with allergic rhinitis [12]. Markedly elevated numbers of activated and degranulated eosinophils were observed in allergic rhinitis patients after allergen exposure [13–15]. In mouse models of allergic lung inflammation, eosinophils can enhance allergic inflammation by promoting the recruitment of T helper type 2 (Th2) cells and interacting with dendritic cells [16, 17]. In addition, eosinophils are able to release preformed Th2 cytokines, such as interleukin- (IL-) 4 and IL-13, to promote type 2 response [18, 19]. Recently, it has been suggested that the activated and pathogenic states of eosinophils, similar to the other immune cell populations that display differences in their phenotype and function, are directly involved in the development of eosinophil-associated diseases, including allergic asthma and eosinophilic esophagitis [20, 21]. However, whether these functional states of eosinophils relate to the disease severity in allergic rhinitis remains unknown.

In this study, we hypothesized that the pathogenic phenotype and function of eosinophils in patients with house dust mite (HDM) allergic rhinitis may alter in relation to the different disease severity statuses. The results of this study will help in understanding the mechanism involved in driving the progression of disease severity of allergic rhinitis and clarify essential issues that may improve the treatment of allergic rhinitis patients.

## 2. Materials and Methods

### 2.1. Patients and Control Subjects

This study was approved by the Ethics Committee of the First Affiliated Hospital, Sun Yat-sen University. Written informed consent was obtained from each participant. Fresh peripheral blood samples and nasal brushing and secretions were collected round 9 am in the morning from all participants.

The diagnosis of allergic rhinitis was evaluated based on the ARIA guideline [1]. The disease severity of allergic rhinitis was classified according to the ARIA guideline and reported as mild and moderate-severe [1]. Briefly, patients with moderate-severe allergic rhinitis had one or more of the following symptoms: sleep disturbance; impairment in daily activities, leisure and/or sports, and school or work; or troublesome symptoms. Patients with mild allergic rhinitis had none of these symptoms. Subjects who had taken oral or nasal corticosteroids or other medications (e.g., antihistamines, antileukotrienes, antibiotics, anticholinergics, or a-adrenergics) for the past 3 months before sample collection were excluded. Other exclusion criteria included (1) sensitization to other inhalant allergens than HDM, such as pollen and fungi; (2) previous treatment with immunotherapy; (3) those with other allergic disease, such as asthma according to the Global Initiative for Asthma guideline; (4) pregnancy or breastfeeding; and (5) those who have had acute or chronic sinusitis; severe immunologic, cardiac, liver, or metabolic disease; tumors; or other chronic infection. The total nasal symptom score (TNSS) was calculated (range: 0–12) by adding up the individual nasal scores including nasal congestion, sneezing, nasal itching, and rhinorrhea, each evaluated using a scale of 0 = none, 1 = mild, 2 = moderate, or 3 = severe [22]. Healthy control subjects had negative skin prick tests to *Dermatophagoides pteronyssinus* (Der p), were without positive specific IgE to common allergens in our region, and had no history of allergy.

### 2.2. Specific IgE Detection

Serum samples were separated by low-speed centrifugation for detection of specific IgE against the local common inhalant allergens using the ImmunoCAP (Phadia, Uppsala, Sweden). Concentration above 0.7 IU/ml was considered a positive result.

### 2.3. Nasal Brushing

A sterile premoistened nylon-flocked brush (HydraFlock 6 in Sterile Standard Flock Swab, Puritan, USA) was used for nasal cell sampling as previously described [23]. Briefly, the brush was placed on the mucosal surface of the medial aspect of the inferior turbinate and turned carefully. No anesthesia was used. After sampling, the brush was immediately placed in a 3 ml plastic tube containing 2 ml phosphate-buffered saline. The brush was then shaken vigorously in the solution and carefully brushed off against the wall of the tube. The cell samples were immediately pelleted via 300 × g centrifugation at 4°C for 5 min, and total cell count was determined manually using a hemocytometer. After cell counting, an aliquot (100 *μ*l) of cells from each sample was then centrifuged onto glass slides using a CytoSpin™ 4 Cytocentrifuge (Thermo Fisher Scientific, Waltham, MA, USA) at 400 × g for 10 min and then rapidly air-dried before immunofluorescence staining. The rest of the cells were immediately subjected to flow cytometry analysis as described below.

### 2.4. Nasal Secretions

Nasal secretions were collected from each participant. Two strips of nasal sponge (hemoX Standard Nasal Dressing; Medtronic Merocel, Jacksonville, Florida, USA) measuring 5 × 10 mm each were inserted in the nostrils (one strip for each nostril) laterally against the inferior turbinate for 5 minutes. Thereafter, the sponge strips were removed and immersed in 500 *μ*l of MILLIPLEX assay buffer (Millipore, Billerica, USA) and then placed in the cup of a cellulose acetate 0.22 mm pore size tube filter (Spin-X Centrifuge Tube Filter, Corning, St. Louis, USA) within an Eppendorf tube and centrifuged for 5 minutes in a cooled centrifuge at 16,000 g. Supernatants were stored at -80°C until analysis.

### 2.5. Immunofluorescence Staining and Eosinophil Counting

Immunofluorescence staining was performed as we previously described [24]. Briefly, after being incubated with 0.1% Triton X-100 and 1% goat serum (Sigma, USA), the slides were incubated overnight at 4°C in the presence of human antieosinophil cationic protein (ECP) (Abcam, ab116017, 1 : 100). Thereafter, each slide was incubated with goat anti-rabbit (Alexa Fluor 488) secondary antibody (Life Technologies, 1 : 500) and then mounted with ProLong Antifade mounting medium with DAPI (Life Technologies). The stained slides were visualized using an Olympus IX51 fluorescence microscope with 40x objective lens. The DAPI- and ECP-positive cells were counted under a fluorescence microscope. A total of 300 cells per sample were evaluated. The total number of eosinophils was calculated from the total number of nasal cells multiplied by the percentage of ECP-positive cells.

### 2.6. Blood Eosinophil Isolation

Eosinophils were isolated by negative selection using the human Eosinophil Isolation Kit (MAC Miltenyi Biotec, Bergisch Gladbach, Germany) following the manufacturer's instructions. Briefly, blood samples were collected from both allergic rhinitis and healthy participants. Blood was layered onto a Ficoll-Hypaque gradient (1.077 g/ml; Pharmacia, Uppsala, Sweden), followed by centrifugation at 450 g, at 20°C for 20 minutes without braking. The layer containing red blood cells (RBCs) and granulocytes was sedimented for 30 minutes in Hank's balanced salt solution (HBSS) buffer containing 4-6% dextran at room temperature. The granulocyte-rich layer with few remaining RBCs was collected and washed once with HBSS buffer. After the hypotonic lysis of residual erythrocytes, eosinophils were separated from noneosinophils by negative immunomagnetic selection using a combination of anti-CD2, anti-CD14, anti-CD16, anti-CD19, anti-CD56, anti-CD123, and anti-CD235a microbeads with a magnetic cell separation column (MAC Miltenyi Biotec, Bergisch Gladbach, Germany). The final eosinophils were collected at a purity of 95% to 99% evaluated by flow cytometry of side scatter and CCR3 expression (Figure S5) and at a viability of more than 98% by trypan blue staining. Cells were resuspended in RPMI medium containing 10% fetal calf serum (FCS) and used immediately.

### 2.7. Eosinophil Degranulation Assay

The eosinophil degranulation assay was performed according to methods described previously with minor modifications [25]. Briefly, 96-well round bottom plates were coated with 3% human serum albumin in Hank's balanced salt solution (HBSS) for 2 hours at 37°C and washed 3 times with HBSS without Ca^2+^ and Mg^2+^. A suspension of isolated eosinophils (200 *μ*l of 5 × 10^5^ cells/ml in RPMI1640 medium with 1% human serum albumin) was incubated in the precoated 96-well plate with 100 ng/ml CCL11 at 37°C for 4 hours. Supernatants were then collected after centrifugation at 13000 × g at 4°C for 5 minutes. The ECP and eosinophil peroxidase (EPX) concentration in supernatants were determined by a specific enzyme-linked immunosorbent assay (ELISA) kit (CUSABIO Life Sciences, Wuhan, China) according to the manufacturer's instructions.

### 2.8. CD4^+^ T Cell Isolation

CD4^+^ T cells were positively purified by using the anti-CD4 antibody-conjugated magnetic nanoparticles (BD Biosciences, San Jose, California, USA) according to the manufacturer's instructions as we previously described [26]. Purity of enriched CD4^+^ T cells was greater than 95% evaluated by flow cytometry. Cells were cultured in RPMI complete medium supplemented with 10% FCS.

### 2.9. CD4^+^ T Cell and Eosinophil Coculture

Coculture of isolated CD4^+^ T cells with purified eosinophils was performed as previously described with some modifications [18]. CD4^+^ T cells were isolated from healthy controls and rested in RPMI complete medium supplemented with 10% FCS overnight at 37°C. Blood eosinophils were purified from healthy controls and subjects with mild and moderate-severe allergic rhinitis. After a wash step, 1 × 10^6^/ml eosinophils were added to an equivalent number of allogeneic CD4^+^ T cells in 96-well round-bottom plates coated with anti-CD3 (5 *μ*g/ml, OKT3, eBioscience) and anti-CD28(1 *μ*g/ml, CD28.2, eBioscience). Cell culture medium consisted of RPMI 1640, 10 mM HEPES, 2 mM l-glutamine, 1% penicillin/streptomycin, 10% FCS (all from Gibco, Grand Island, New York, USA), and 10 ng/ml recombinant human granulocyte-macrophage colony-stimulating factor (GM-CSF) (PeproTech Inc., Rocky Hill, New Jersey, USA). After 5 days, a cell stimulation cocktail composed of phorbol 12-myristate 13-acetate (PMA) (81 nM), ionomycin (1.34 mM), brefeldin A (10.6 mM), and monensin (2 mM) (eBioscience, San Diego, California, USA) was added for induction and subsequent intracellular detection of cytokines. After 5 hours, cells were harvested and further analyzed by flow cytometry as described below.

### 2.10. Flow Cytometry

For peripheral blood eosinophil analysis, blood granulocytes were first isolated as described above. Single-cell suspensions were stained with Fixable Viability Dye eFluor 450 (eBioscience, San Jose, California, USA) to exclude dead cells. To block nonspecific staining, cells were incubated with human TruStain FcX (BioLegend, San Diego, California, USA) for 10 min on ice. For surface staining, cells were incubated with specific primary antibodies (Table 1) for 30 min at 4°C. For intracellular staining, cells were washed twice after surface staining and permeabilized using Transcription Factor Fix/Perm Buffer (BD Bioscience) for 30 min at room temperature. Antibodies specific for intracellular cytokines were diluted in Perm/Wash buffer (BD Bioscience) and incubated for 30 min on ice. Isotype controls were used as negative controls. Fluorescence minus one control for each fluorochrome was used to set the gate for positive populations. The stained cells were analyzed by using a Gallios flow cytometer (Beckman Coulter, Brea, California, USA), and data were analyzed with FlowJo software (TreeStar, Ashland, Oregon, USA).

### 2.11. Cytokine Assays

The concentrations of IL-4, IL-5, IL-13, IL-17A, and IFN-*γ* in the blood serum and nasal secretion were detected by using Luminex-based Multiplex kits (Millipore) according to the manufacturer's protocol [24]. The levels of ECP (CUSABIO Life Sciences, Wuhan, China) and EPX (CUSABIO Life Sciences, Wuhan, China) were measured using commercially available ELISA kits. For convenient analysis, all values below the detectable limit were considered zero [24]. Detection limits for the Luminex-based assay and ELISA assay were listed in Table 2.

### 2.12. Statistical Analysis

All statistical analyses were performed with GraphPad Prism Software 7.0 (GraphPad Software, La Jolla, California, USA). Expression data are presented in dot plots unless specifically indicated. Symbols represent individual samples, horizontal bars represent median, and error bars show interquartile range. A Kruskal-Wallis *H* test was used to assess significant intergroup variability among more than 2 groups and a Mann-Whitney *U* 2-tailed test was used for between-group comparison. Cell culture data are expressed as mean ± SEM and analyzed by unpaired Student *t*-test and one-way analysis of variance (ANOVA) unless specifically stated. For categorical data, chi-squared test or Fisher's exact test was performed. Spearman's rank correlation analysis was used to analyze the associations. *P* value less than 0.05 was considered statistically significant.

## 3. Results and Discussion

### 3.1. Demographic and Clinical Characteristics

From January 2018 to December 2018, 10 mild and 21 moderate-severe allergic rhinitis patients with HDM allergy and 10 healthy subjects without allergy with age ranging from 20 to 31 years participated in the cohort study (Table 3). Based on the ARIA classification [1], all subjects with moderate-severe AR were persistent AR, and only 2 of 10 mild AR subjects were intermittent AR (Table 3). Moderate-severe patients had a median TNSS of 7, whereas mild patients had a median TNSS of 1 (Figure S1A). As expected, the levels of Der p-specific IgE in serum were significantly higher in the moderate-severe patients than in the mild patients (Table 3). There was no difference in sex, age, and smoking history between the three groups studied (Table 3).

### 3.2. The Clinical Severity of Allergic Rhinitis Correlates with the Levels of Eosinophils in the Blood and Nose

**Figure 1:**
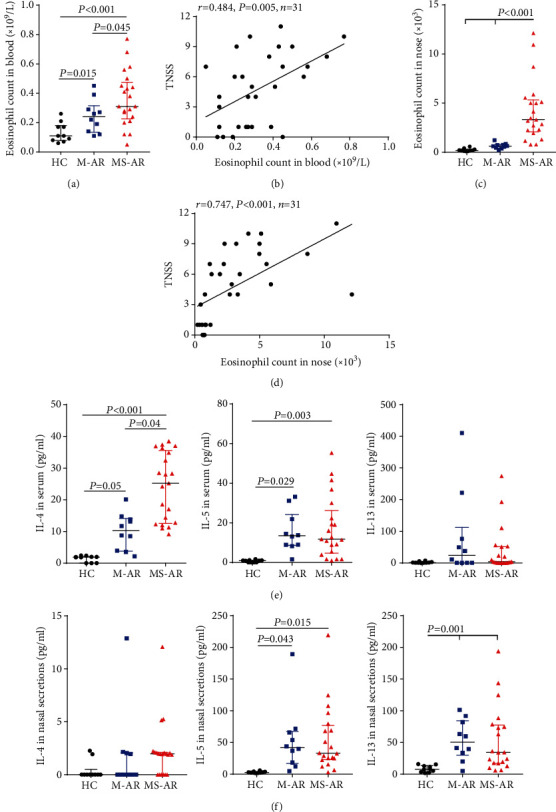
The clinical severity of allergic rhinitis correlates with the levels of eosinophils in the blood and nose. Eosinophil count in the peripheral blood (a) and nose (nasal brushing) (b) of healthy controls, mild allergic rhinitis, and moderate-severe allergic rhinitis. Positive correlation of TNSS with the blood (b) and nasal (d) eosinophil counts in allergic rhinitis. Levels of Th2-related cytokines IL-4, IL-5, and IL-13 in the peripheral blood (e) and nasal secretions (f) of healthy controls and mild and moderate-severe allergic rhinitis. HC: healthy controls; M-AR: mild allergic rhinitis; MS-AR: moderate-severe allergic rhinitis.

We found that the eosinophil levels in the blood were significantly higher in mild and moderate-severe allergic rhinitis compared to healthy controls. In addition, the moderate-severe patients had higher levels of blood eosinophils than the mild patients (Figure 1(a)). Interestingly, increased numbers of total nasal cells and eosinophils were only observed in the moderate-severe patients (Figure 1(c) and Figure S1B-C). Furthermore, the TNSS was significantly correlated with eosinophil counts in the blood and nose in patients with allergic rhinitis (*r* = 0.484 and 0.747, respectively) (Figures 1(b) and 1(d)).

We also measured the levels of Th2 cytokines in serum and nasal secretions. The concentrations of IL-4 and IL-5 in serum and IL-5 and IL-13 in nasal secretions were significantly increased in subjects with mild and moderate-severe allergic rhinitis compared to healthy controls (Figures 1(e) and 1(f)). However, we found that only the moderate-severe patients had significantly higher levels of IL-4 in serum than the mild patients (Figure 1(e)). We did not find significant differences in the IL-13 levels in serum and IL-4 levels in nasal secretions between the three groups (Figures 1(e) and 1(f)). In addition, there was no significant difference in the levels IL-17A and IFN-*γ* in blood and nasal secretions between the three groups (data not shown).

### 3.3. The Proportion of Activated Eosinophils Is Increased in Moderate-Severe Allergic Rhinitis

**Figure 2:**
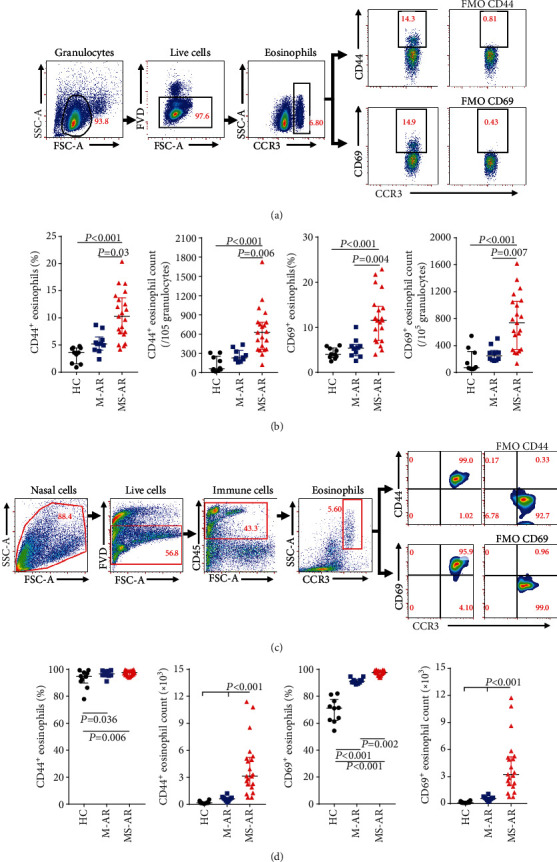
The numbers of activated eosinophils are increased in moderate-severe allergic rhinitis. Gating strategies used to characterize the activated status of eosinophils in the peripheral blood (a) and nose (c). Percentages and absolute numbers of CD44- and CD69-expressing eosinophils in the peripheral blood (b) and nose (d). Data are presented as median with interquartile range. HC: healthy controls; M-AR: mild allergic rhinitis; MS-AR: moderate-severe allergic rhinitis; FMO: fluorescence minus one control.

We next analyzed the activation status of eosinophils in allergic rhinitis. Figures 2(a) and 2(c) show the gating strategies used to characterize the profile of activated eosinophils in the blood and nose. Surprisingly, we found that the percentages and counts of CD44^+^ and CD69^+^ eosinophils in the blood were only increased in the moderate-severe patients compared to the healthy controls. Most eosinophils in nasal brushes expressed both CD44 and CD69, although the percentages of CD44^+^ and CD69^+^ eosinophils were significantly increased both in the mild and moderate-severe patients compared to healthy controls (Figure 2(d)). However, the counts of CD44^+^ and CD69^+^ eosinophils in nasal brushes were increased in the moderate-severe patients, but not in the mild patients (Figure 2(d)). The TNSS was significantly positively correlated with the counts of CD44^+^ and CD69^+^ eosinophils in the blood and nose (Figure S3A-B).

### 3.4. Pathogenic Eosinophils Are Elevated in Patients with Moderate-Severe Allergic Rhinitis

**Figure 3:**
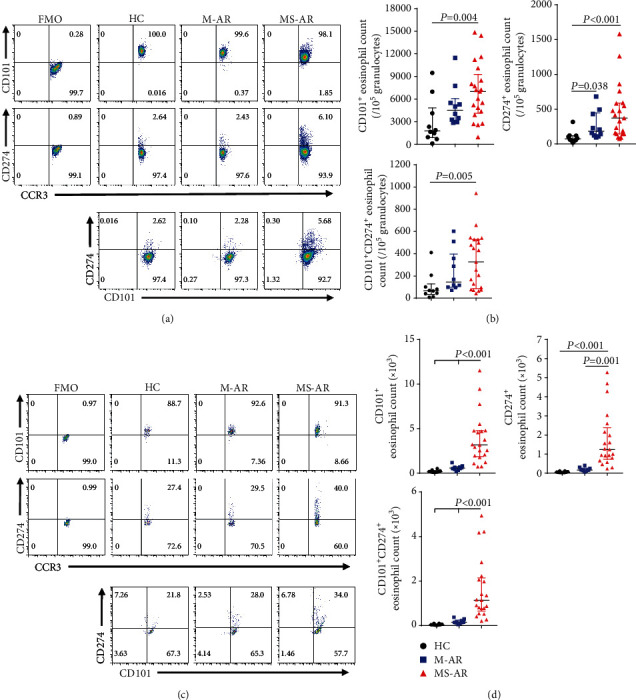
Pathogenic eosinophils are elevated in patients with moderate-severe allergic rhinitis. (a, c) Representative flow plots for identifying CD101^+^ and CD274^+^ eosinophils in the peripheral blood (a) and nose (c) of healthy controls, mild allergic rhinitis, and moderate-severe allergic rhinitis. All samples were first gated on live eosinophils. (b, d) Absolute numbers of CD101^+^, CD274^+^, and CD101^+^CD274^+^ eosinophils in the peripheral blood (b) and nose (d). HC: healthy controls; M-AR: mild allergic rhinitis; MS-AR: moderate-severe allergic rhinitis; FMO: fluorescence minus one control.

Recent studies have revealed that CD101 and CD274 are markers of mature and activated pathogenic eosinophils in asthmatics and eosinophilic esophagitis [21, 27, 28]; we hence examined the CD101^+^ and CD274^+^ eosinophils in allergic rhinitis. In line with other allergic diseases [21], most eosinophils in the peripheral blood and in nasal brushes were CD101^+^ (Figure S2A-B). However, we found that the counts of CD101^+^ eosinophils in the blood and nose were only increased in the moderate-severe patients compared to the healthy controls (Figures 3(b) and 3(d)). In addition, although both mild and moderate-severe patients had more CD274^+^ eosinophils in the blood than healthy controls (Figures 3(b) and 3(d)), their percentages in the blood and nose as well as their counts in nasal brushes were only increased in the moderate-severe patients (Figures 3(b) and 3(d) and Figure S2A-B). Surprisingly, we also found that the percentages and counts of CD101^+^CD274^+^ double-positive eosinophils in the blood and nose were only increased in the moderate-severe patients compared to the healthy controls (Figures 3(b) and 3(d) and Figure S2A-B). Furthermore, the TNSS was significantly positively correlated with the CD101^+^CD274^+^ double-positive eosinophils in the blood and nose (Figure S3A-B).

### 3.5. Levels of Eosinophil Progenitors in Peripheral Blood Are Increased in Patients with Moderate-Severe Allergic Rhinitis

**Figure 4:**
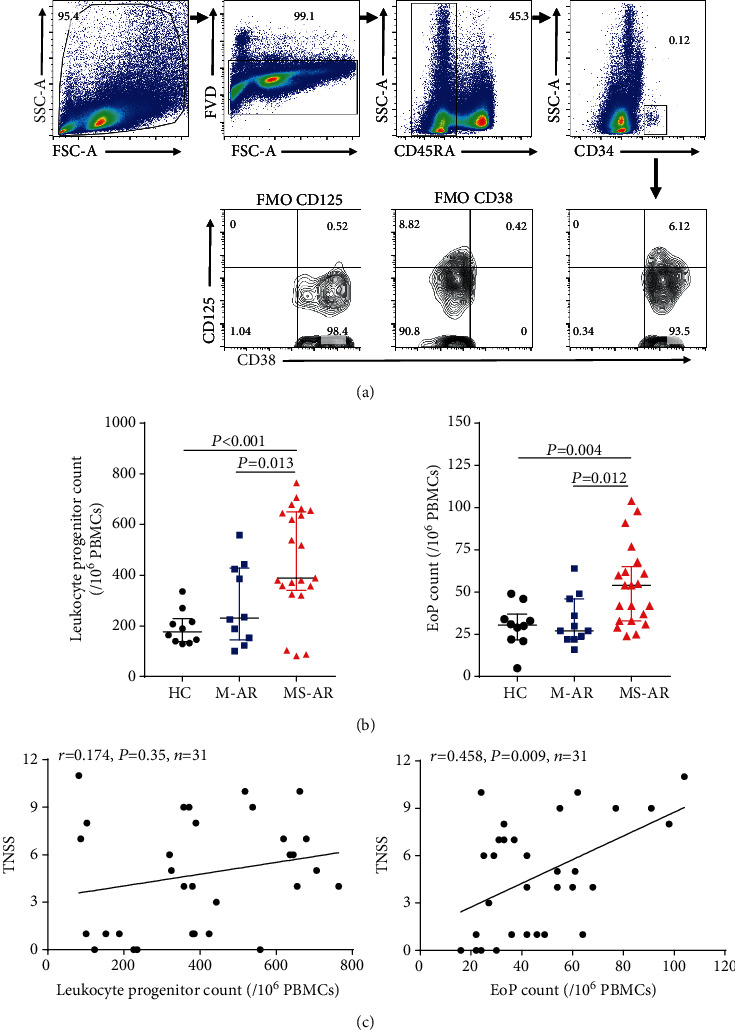
Levels of eosinophil progenitors in the peripheral blood are increased in patients with moderate-severe allergic rhinitis. (a) Gating strategy for of leukocyte progenitors (live CD45RA-CD34^+^ cells) and eosinophil progenitors (live CD45RA-CD34^+^CD125^+^CD38^+^ cells) in the peripheral blood. (b) Levels of leukocyte progenitors and eosinophil progenitors in the peripheral blood of healthy controls, mild allergic rhinitis, and moderate-severe allergic rhinitis. Progenitor levels are expressed in absolute numbers per one million PBMCs. (b) Correlation of leukocyte progenitor and eosinophil progenitor absolute numbers in patients with allergic rhinitis in the peripheral blood with TNSS. HC: healthy controls; M-AR: mild allergic rhinitis; MS-AR: moderate-severe allergic rhinitis; PBMCs: peripheral blood mononuclear cells; FMO: fluorescence minus one control; EoP: eosinophil progenitor.

Previous studies have suggested a role for progenitors in allergic diseases, including bronchial asthma and allergic rhinitis [29, 30]; we then evaluated the numbers of leukocyte progenitor and eosinophil progenitor cells in the peripheral blood of the subjects studied. Interestingly, the absolute numbers of leukocyte progenitors and eosinophil progenitors in the blood were only increased in the moderate-severe subjects compared with healthy controls (Figures 4(a) and 4(b)). The levels of circulating eosinophil progenitors were significantly positively correlated with TNSS (Figure 4(c), *r* = 0.458). However, there was no significant correlation between the leukocyte progenitor counts and TNSS (Figure 4(c)).

### 3.6. Blood Eosinophils from Subjects with Moderate-Severe Allergic Rhinitis Produce More Granule Proteins and Had a Pro-Th2 Capacity

**Figure 5:**
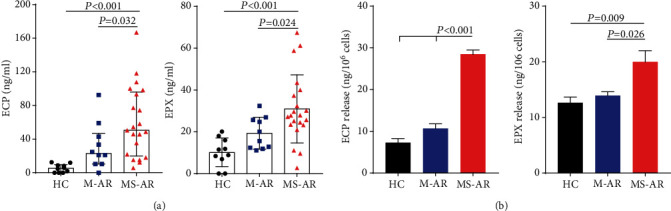
Blood eosinophils from subjects with moderate-severe allergic rhinitis produce more granule proteins. (a) Serum ECP and EPX levels in healthy controls, mild allergic rhinitis, and moderate-severe allergic rhinitis. (b) ECP and EPX release from blood eosinophils induced by CCL11. ECP: eosinophil cationic protein; EPX: eosinophil peroxidase; HC: healthy controls; M-AR: mild allergic rhinitis; MS-AR: moderate-severe allergic rhinitis.

We next measured the levels of ECP and EPX, two important eosinophilic granule proteins involved in the pathogenesis of allergic rhinitis, in the serum and found that, consistent with the levels of pathogenic eosinophils, the serum ECP and EPX levels were only significantly increased in the moderate-severe patients compared with the healthy controls, although there were trends toward increases in the mild patients (Figure 5(a)). In addition, the serum ECP levels positively correlated with TNSS and the counts of CD44^+^, CD69^+^, and CD101^+^CD274^+^ eosinophils in the blood (*r* = 0.593, 0.47, 0.414, and 0.386, respectively) (Figure S4A). Positive correlations were also observed between the serum EPX levels and TNSS and the blood CD101^+^CD274^+^ eosinophil counts (*r* = 0.425 and 0.403, respectively) (Figure S4B). There was a trend toward a correlation between the serum EPX levels and the blood CD69^+^ eosinophil counts (*r* = 0.329, *P* = 0.071) (Figure S4B).

We further performed an eosinophil degranulation experiment to compare the potential of eosinophils in the production of eosinophilic granule proteins between the three groups. The demographics of the subjects used in the in vitro experiment are presented in Table 4. As a result, we found that blood eosinophils derived from the moderate-severe patients produced more ECP and EPX induced by CCL11 compared with those from the mild patients and healthy controls (Figure 5(b)). Interestingly, there was no significant difference in ECP and EPX production between eosinophils derived from the mild patients and healthy controls (Figure 5(b)).

**Figure 6:**
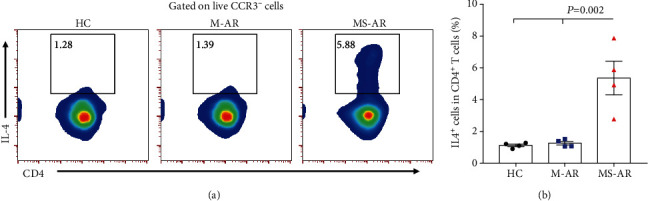
Blood eosinophils derived from moderate-severe allergic rhinitis have a pro-Th2 capacity. (a) Representative flow plots and (b) quantification of IL-4 expression by CD4^+^ T cells obtained from healthy controls after coculture with blood eosinophils from healthy controls, mild allergic rhinitis, and moderate-severe allergic rhinitis for five days. HC: healthy controls; M-AR: mild allergic rhinitis; MS-AR: moderate-severe allergic rhinitis.

Studies have showed that eosinophils could promote type 2 response under certain circumstances [18, 31]. It is of great interest to know whether eosinophils from allergic rhinitis have a pro-Th2 capacity. We thus performed a coculture of CD4^+^ T cells with blood eosinophils derived from the healthy controls, mild patients, and moderate-severe patients in the presence of anti-CD3/28 for five days. The results showed that CD4^+^ T cells produced more IL-4 only when cocultured with eosinophils from the moderate-severe patients compared with those from the healthy controls and mild patients (Figures 6(a) and 6(b)). There was no significant difference in the expressions of IL-5, IL-13, IL-17A, and IFN-*γ* in CD4^+^ T cells between cocultures with eosinophils from the three groups (data not shown).

## 4. Discussion

The link between eosinophils and type 2-related diseases such as bronchial asthma and allergic rhinitis has been well established [10]. Although ever-increasing knowledge is gained on the function of eosinophils, it is still not known exactly when and how they relate to the development of allergic disease severity. Previous studies have shown that the numbers of eosinophils correlated with the clinical symptoms, such as nasal hyperactivity and nasal congestion, in patients with allergic rhinitis [12, 32]. In addition, another study found significant increases in the total number of eosinophils and the number of activated eosinophils in the nose of patients with grass pollen-allergic rhinitis after 2 weeks of daily challenge with allergen [33]. In the present study, we extended previous work to analyze the activated and pathogenic phenotypes of eosinophils in patients with HDM-allergic rhinitis according to their different disease severity statuses. The novel finding is that the levels of activated (CD44^+^ and CD69^+^) and pathogenic (CD101^+^CD274^+^) eosinophils were particularly higher in subjects with moderate-severe HDM-allergic rhinitis compared to the mild patients and healthy controls and were positively correlated with the TNSS and serum ECP levels in subjects with allergic rhinitis. Moreover, blood eosinophils derived from the moderate-severe patients exhibited a stronger capacity in producing ECP and EPX induced by CCL11 and promoting Th2 differentiation than those from the mild patients and healthy controls. To the best of our knowledge, these data provide the first evidence to reveal the difference of the functional state of eosinophils in patients with allergic rhinitis in relation to disease severity. Importantly, our study first showed a stronger capability of eosinophils in promoting Th2 response in moderate-severe allergic rhinitis patients, suggesting a novel possible mechanism for eosinophils in driving the progression of disease severity of allergic rhinitis.

CD44 and CD69 are two major activation markers of human eosinophils, although there were studies suggesting they represent different types of activation [34, 35]. In the present study, we found that most eosinophils in nasal brushes were CD44 and CD69 positive. These results are largely consistent with previous studies showing that eosinophils obtained from the bronchoalveolar lavage fluid of patients with eosinophilic airway diseases expressed higher levels of CD44 and CD69 than those in the peripheral blood [36, 37]. Together, these findings indicate that eosinophils in airways were more activated than those in the blood. It has been reported that CD44 and CD69 expressions on eosinophils were upregulated in BAL and sputum after lung allergen challenge [38, 39]. In line with these studies, we found that the percentages and counts of blood CD44^+^ and CD69^+^ eosinophils as well as the counts of nasal CD44^+^ and CD69^+^ eosinophils were only significantly increased in the moderate-severe patients, suggesting that blood eosinophils become activated and extravasate into the nose when subjects encounter allergen stimulation.

Recently, associations between specific surface phenotypes and functions of eosinophils have been demonstrated in mouse models of allergic inflammation. An eosinophil subset expressing a high level of CD101 was shown to promote inflammatory response in the lungs of mice following HDM challenge [27]. More recently, studies reported that CD101^+^CD274^+^ eosinophils were induced in the blood of patients with eosinophilic esophagitis and allergic asthma, and their levels were positively associated with disease severity [21, 40]. In addition, CD101^+^ CD274^+^ eosinophils were responsible for IL-18-induced allergic intestine inflammation in mice, demonstrating that CD101 and CD274 are markers of pathogenic eosinophils [28]. Consistent with these studies, our study also found that the majority of eosinophils expressed CD101. However, the counts of CD101^+^ and CD101^+^CD274^+^ eosinophils in both the peripheral blood and nose were only increased in the moderate-severe patients compared with the mild patients and healthy controls. In addition, a positive correlation between TNSS and the counts of blood and nasal CD101^+^CD274^+^ eosinophils was observed in subjects with allergic rhinitis. Taken together, these findings suggest that the eosinophils of subjects with moderate-severe allergic rhinitis have enhanced pathogenicity, and those pathogenic eosinophils might be involved in driving the progression of disease severity of allergic rhinitis. Moreover, it will be of great interest to decipher the distinct mechanism by which eosinophils drive the symptoms associated with mild allergic rhinitis. Future studies using a high-throughput RNA-seq approach to characterize the transcriptional profiling of eosinophils might be helpful in addressing this issue.

The pathogenic role of eosinophils in the moderate-severe subjects was also supported by the in vitro function experiments of our study showing that blood eosinophils obtained from the moderate-severe patients produced more ECP and EPX induced by CCL11 with a capability of promoting Th2 response in vitro compared with those from the mild patients and healthy controls (Figures 5 and 6). ECP and EPX, as well as major basic protein and eosinophil-derived neurotoxin, are cationic proteins enriched in human eosinophilic granules [41]. The production and biological activities of these cationic proteins have been a focus of studying the effector functions of eosinophils. It has been reported that the release of eosinophil granule proteins in asthmatic lungs was linked with the pathogenesis of lung remodeling and airway hyperresponsiveness [42–44]. Moreover, recent studies have shown that Th2 cytokine IL-13 production of eosinophils is necessary for the pro-Th2 functions of eosinophils in eosinophilic lung pathologies [45, 46]. Together, these findings suggest that eosinophil activities likely contribute to allergic inflammation at many levels in the moderate-severe allergic rhinitis patients.

Eosinophil progenitor cells migrate from the bone marrow and can differentiate peripherally to provide an ongoing source of mature eosinophils in the inflamed airways of asthmatics [47]. A growing body of evidence has emerged suggesting an important role for eosinophil progenitors in allergic inflammation in both human and animal models [48]. Studies have reported that eosinophil progenitors were increased in the peripheral blood in patients with asthmatics and allergic rhinitis. Moreover, the levels of eosinophil progenitors were elevated in the lungs and sputum of patients with asthma after allergen challenge [30, 49]. However, no clinical studies to date have examined the relationship of eosinophil progenitors in the peripheral blood with the disease severity of allergic rhinitis. Our results revealed that the absolute numbers of leukocyte progenitors and eosinophil progenitors in the blood were only increased in the moderate-severe patients (Figures 4(a) and 4(b)). Furthermore, a significant positive correlation was found between the levels of circulating eosinophil progenitors and TNSS. These findings suggest a direct contribution of eosinophil progenitors to blood eosinophilia during the progression of disease severity of allergic rhinitis, thus advancing the concept of the eosinophil progenitors as a clinically useful biomarker for disease severity in allergic rhinitis.

Several possible explanations might be responsible for the discrepancy in IL-4 and IL-13 levels between serum nasal secretions (Figures 1(e)–1(f)). First, the discrepancy may reflect the different effector function of IL-4 and IL-13 between the blood and upper airway. For example, IL-4 mainly functions to induce B-cell class switching to IgE in germinal centres, whereas IL-13 induces goblet cell hyperplasia and mucus hypersecretion in the airway. Second, there appears to be a general increase of IL-4 in the nasal serum and IL-13 in the serum in patients with AR; therefore, methodologic reasons, such as low detection sensitivity of Luminex-based Multiplex and relatively small sample size of the present study, could not be ruled out. Larger studies are warranted in the future.

A clear limitation of the present study is that we only conducted an in vitro study. Whether eosinophils in the moderate-severe patients actually have a higher pathogenicity is still unknown. This limitation may be addressed in the future work through animal models of allergic airway inflammation. In addition, the sample size is relatively small, since we did not observe several expected correlations such as between serum EPX levels and CD44^+^ and CD69^+^ eosinophil count in the blood (Figure 4). It also should be noted that correlations between the serum ECP and EPX levels and the blood CD69^+^ and CD101^+^CD274^+^ eosinophil counts were relatively week although *P* values were less than 0.05 (Figure S4). Moreover, because of the largely descriptive nature of our study, one should be cautious when interpreting and generalizing our findings. These limitations could be well addressed by large, prospective longitudinal studies.

In conclusion, our study reveals that elevated levels of activated and pathogenic eosinophils are a feature of moderate-severe allergic rhinitis and associated with higher production of ECP, EPX, and IL-4 in the peripheral blood.

## Figures and Tables

**Table 1:** Antibodies used for flow cytometry.

Antibody	Parameter	Clone ID	Source	Isotype	Manufacturer	Dilution
CCR3	PerCP/cyanine5.5	5EB	Mouse	IgG2b, *κ*	BioLegend	1 : 100
CD44	FITC	BJ18	Mouse	IgG1, *κ*	BioLegend	1 : 100
CD69	PE	FN50	Mouse	IgG1, *κ*	BioLegend	1 : 100
CD101	PE/Cy7	BB27	Mouse	IgG1, *κ*	BioLegend	1 : 100
CD274	APC	29E.2A3	Mouse	IgG2b, *κ*	BioLegend	1 : 100
CD45	APC/cyanine7	HI30	Mouse	IgG1, *κ*	BioLegend	1 : 100
Siglec-8	PE	7C9	Mouse	IgG1, *κ*	BioLegend	1 : 100
CD34	FITC	561	Mouse	IgG2a, *κ*	BioLegend	1 : 100
CD38	PE/Cy7	HIT2	Mouse	IgG1, *κ*	BioLegend	1 : 100
CD125	PE	A14	Mouse	IgG1, *κ*	BD	1 : 100
CD45RA	APC	HI100	Mouse	IgG2b, *κ*	BioLegend	1 : 100
CD4	APC/cyanine7	RPA-T4	Mouse	IgG1, *κ*	BioLegend	1 : 200
IL-4	PE	8D4-8	Mouse	IgG1, *κ*	BioLegend	1 : 100
IL-4	PerCP/cyanine5.5	8D4-8	Mouse	IgG1, *κ*	BD	1 : 100
IL-5	FITC	# 9906	Mouse	IgG1, *κ*	R&D	1 : 100
IL-17A	Alexa Fluor® 647	BL168	Mouse	IgG1, *κ*	BioLegend	1 : 100
IFN-*γ*	PE/Cy7	4S.B3	Mouse	IgG1, *κ*	BioLegend	1 : 100

**Table 2:** Detection limits for cytokine measurement.

Target	Detection limit	Manufacturer
IL-4	1.83 pg/ml	Millipore Luminex-based multiplex
IL-5	0.49 pg/ml	Millipore Luminex-based multiplex
IL-13	0.24 pg/ml	Millipore Luminex-based multiplex
IL-17A	0.73 pg/ml	Millipore Luminex-based multiplex
IFN-*γ*	0.61 pg/ml	Millipore Luminex-based multiplex
ECP	1.56 ng/ml	CUSABIO Life Sciences
EPX	3.12 ng/ml	CUSABIO Life Sciences

**Table 3:** Demographic summary of subjects in the cohort study.

	Healthy controls	Mild AR	Moderate-severe AR	*P* value
Total subjects, *n*	10	10	21	—
Gender, male/female	4/6	7/3	11/10	0.399
Age (years)	24 (22, 25)	25 (22, 26.25)	23 (22, 25.5)	0.552
History of smoking (%)	1 (10)	0	0	0.204
Intermittent/persistent	/	2/8	0/21	—
SPT to Der p	Negative	Positive	Positive	—
Total IgE (KU/l)	53.4 (28.7, 78.1)	99.9 (81.7, 118.1)	137.6 (82.2, 193)	0.000
Specific IgE to Der p (KU/l)	0.07 (0.04, 0.15)	23.0 (7.48, 47.53)	65.0 (13.65, 99.2)	0.000

For continuous variables, data are expressed by medians and interquartile ranges. AR: allergic rhinitis; SPT: skin prick test; Der p: *Dermatophagoides pteronyssinus*; Ig: immunoglobulin.

**Table 4:** Demographic summary of subjects used in the in vitro eosinophil function study.

	Healthy controls	Mild AR	Moderate-severe AR	*P* value
Total subjects, *n*	4	4	4	—
Gender, male/female	2/2	2/2	2/2	—
Age (years)	22.5 (22, 25.25)	25.5 (22.75, 26.75)	25.5 (22, 30.5)	0.665
History of smoking (%)	0	0	0	—
SPT to Der p	Negative	Positive	Positive	—
Total IgE (KU/l)	29.3 (12.1, 46.5)	95.2 (87.5, 102.9)	180.1 (113.8, 246.4)	0.001
Specific IgE to Der p (KU/l)	0.05 (0.04, 0.06)	27.8 (10.95, 43.98)	76.35 (32.68, 94.3)	0.001

For continuous variables, data are expressed by medians and interquartile ranges. AR: allergic rhinitis; SPT: skin prick test; Der p: *Dermatophagoides pteronyssinus*; Ig: immunoglobulin.

## Data Availability

The data used to support the findings of this study are available from the corresponding author upon request.

## Supplementary Materials

Supplementary Materials Figure S1: increased TNSS, total cell count, and eosinophils in subjects with moderate-severe allergic rhinitis. (a) TNSS. (b) Total cell count in the nose by nasal brushing. (c) Representative images of immunofluorescence staining showing that eosinophils (ECP+ cells, green) in the nose are increased in the moderate-severe patients. TNSS: total nasal symptom score; HC: healthy controls; M-AR: mild allergic rhinitis; MS-AR: moderate-severe allergic rhinitis; ECP: eosinophil cationic protein; DAPI: 4′,6-diamidino-2-phenylindole (blue). Figure S2: percentages of CD101-, CD274-, and both CD101- and CD274-expressing eosinophils in the peripheral blood (a) and nose (b). HC: healthy controls; M-AR: mild allergic rhinitis; MS-AR: moderate-severe allergic rhinitis. Figure S3: relationship between TNSS and eosinophil subsets in patients with allergic rhinitis. Correlation of TNSS with CD44+, CD69+ and CD101+CD274+ eosinophil count in the blood (a) and nose (b). TNSS: total nasal symptom score. Figure S4: relationship between eosinophil granule proteins and eosinophil subsets in patients with allergic rhinitis. Correlation of serum ECP (a) and EPX (b) concentrations with TNSS, total, CD44+, CD69+, and CD101+CD274+ eosinophil count in the blood. TNSS: total nasal symptom score; ECP: eosinophil cationic protein; EPX: eosinophil peroxidase. Figure S5: purity of eosinophils isolated from the peripheral blood. Representative flow cytometry plots showing that the purity of eosinophils was greater than 96%.
