# Supplementary material for: Elevated Levels of Activated and Pathogenic Eosinophils Characterize Moderate-Severe House Dust Mite Allergic Rhinitis
Source: J Immunol Res. 2020 Aug 13;2020:8085615. doi: 10.1155/2020/8085615 (PMC7443015; doi:10.1155/2020/8085615)
Supplement: Supplementary Materials — Figure S1: increased TNSS, total cell count, and eosinophils in subjects with moderate-severe allergic rhinitis. (a) TNSS. (b) Total cell count in the nose by nasal brushing. (c) Representative images of immunofluorescence staining showing that eosinophils (ECP+ cells, green) in the nose are increased in the moderate-severe patients. TNSS: total nasal symptom score; HC: healthy controls; M-AR: mild allergic rhinitis; MS-AR: moderate-severe allergic rhinitis; ECP: eosinophil cationic protein; DAPI: 4′,6-diamidino-2-phenylindole (blue). Figure S2: percentages of CD101-, CD274-, and both CD101- and CD274-expressing eosinophils in the peripheral blood (a) and nose (b). HC: healthy controls; M-AR: mild allergic rhinitis; MS-AR: moderate-severe allergic rhinitis. Figure S3: relationship between TNSS and eosinophil subsets in patients with allergic rhinitis. Correlation of TNSS with CD44+, CD69+ and CD101+CD274+ eosinophil count in the blood (a) and nose (b). TNSS: total nasal symptom score. Figure S4: relationship between eosinophil granule proteins and eosinophil subsets in patients with allergic rhinitis. Correlation of serum ECP (a) and EPX (b) concentrations with TNSS, total, CD44+, CD69+, and CD101+CD274+ eosinophil count in the blood. TNSS: total nasal symptom score; ECP: eosinophil cationic protein; EPX: eosinophil peroxidase. Figure S5: purity of eosinophils isolated from the peripheral blood. Representative flow cytometry plots showing that the purity of eosinophils was greater than 96%. [file 8085615.f1.pdf]

Supplementary Materials

Figure S1. Increased TNSS, total cell count and eosinophils in subjects with moderate-severe allergic rhinitis. (A) TNSS. (B) Total cell count in nose by nasal brushing. (C) Representative images of immunofluorescence staining showing eosinophils (ECP^+ cells, green) in nose are increased in the moderate-severe patients. TNSS, total nasal symptom score. HC, healthy controls. M-AR, mild allergic rhinitis. MS-AR, moderate-severe allergic rhinitis. ECP, eosinophil cationic protein. DAPI, 4′,6-diamidino-2-phenylindole (blue).

Figure S2. Percentages of CD101-, CD274-, and both CD101- and CD274-expressing eosinophils in peripheral blood (A) and nose (B). HC, healthy controls. M-AR, mild allergic rhinitis. MS-AR, moderate-severe allergic rhinitis.

Figure S3. Relationship between TNSS and eosinophil subsets in patients with allergic rhinitis. Correlation of TNSS with $CD44^+$, $CD69^+$ and $CD101^+CD274^+$ eosinophil count in blood (A) and nose (B). TNSS, total nasal symptom score.

Figure S4. Relationship between eosinophil granule proteins and eosinophil subsets in patients with allergic rhinitis. Correlation of serum ECP (A) and EPX (B) concentrations with TNSS, total, $CD44^+$, $CD69^+$ and $CD101^+CD274^+$ eosinophil count in blood. TNSS, total nasal symptom score. ECP, eosinophil cationic protein. EPX, eosinophil peroxidase.

Figure S5. Purity of eosinophils isolated from peripheral blood. Representative flow-cytometry plots showing the purity of eosinophils were greater than 96%.

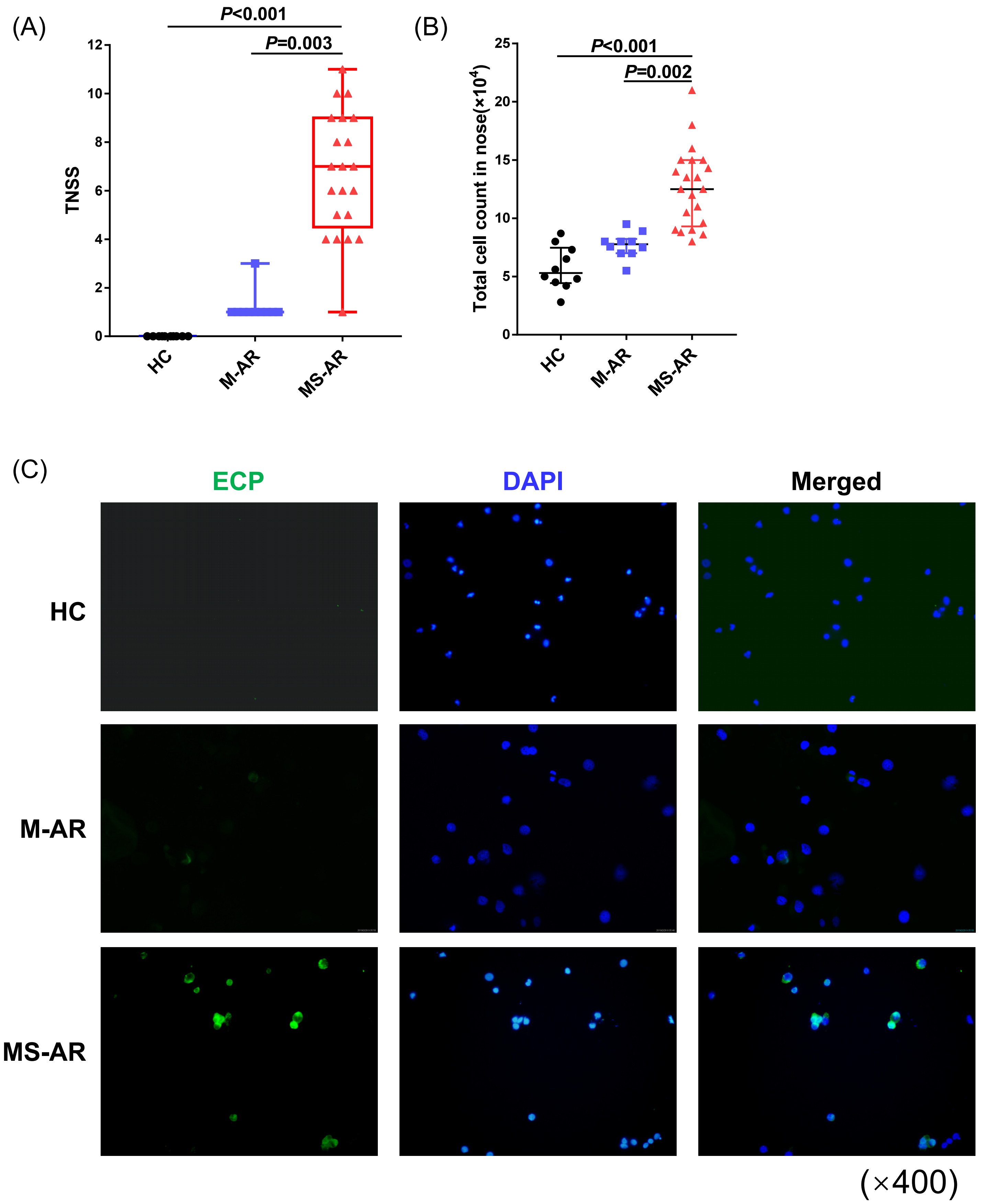

(A)
P<0.001
P=0.003
TNSS
12
10
8
6
4
2
0
HC
M-AR
MS-AR
(B)
P<0.001
P=0.002
Total cell count in nose(×10⁴)
25
20
15
10
5
0
HC
M-AR
MS-AR
(C)
ECP
DAPI
Merged
HC
M-AR
MS-AR
(×400)

Figure S1

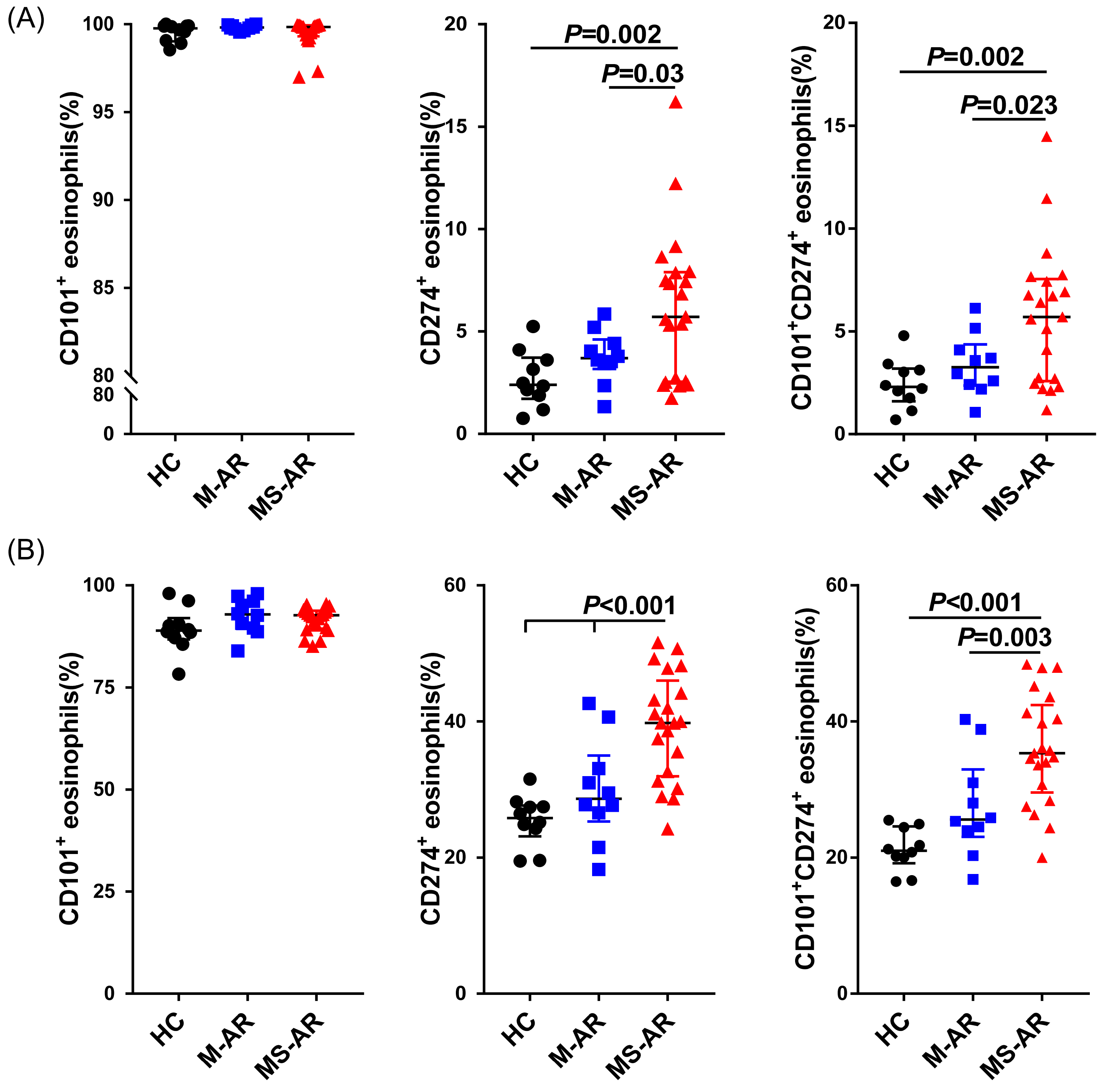

(A)
CD101+ eosinophils(%)
100
95
90
85
80
80
0
HC
M-AR
MS-AR
CD274+ eosinophils(%)
20
15
10
5
0
P=0.002
P=0.03
CD101+CD274+ eosinophils(%)
P=0.002
P=0.023
(B)
CD101+ eosinophils(%)
100
75
50
25
0
CD274+ eosinophils(%)
60
40
20
P<0.001
CD101+CD274+ eosinophils(%)
P<0.001
P=0.003

Figure S2

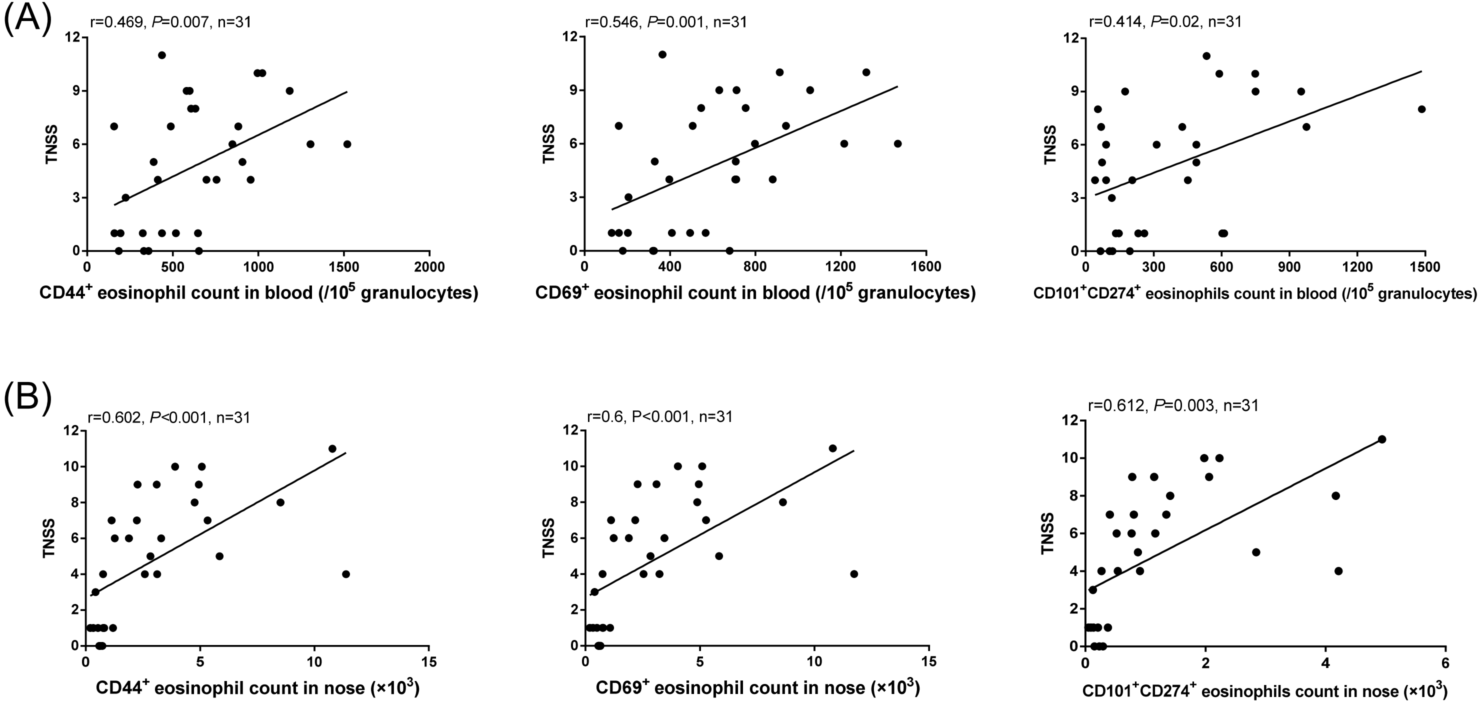
(A)
r=0.469, P=0.007, n=31
TNSS
CD44+ eosinophil count in blood (/10^5 granulocytes)
r=0.546, P=0.001, n=31
TNSS
CD69+ eosinophil count in blood (/10^5 granulocytes)
r=0.414, P=0.02, n=31
TNSS
CD101+CD274+ eosinophils count in blood (/10^5 granulocytes)
(B)
r=0.602, P<0.001, n=31
TNSS
CD44+ eosinophil count in nose (×10^3)
r=0.6, P<0.001, n=31
TNSS
CD69+ eosinophil count in nose (×10^3)
r=0.612, P=0.003, n=31
TNSS
CD101+CD274+ eosinophils count in nose (×10^3)

Figure S3

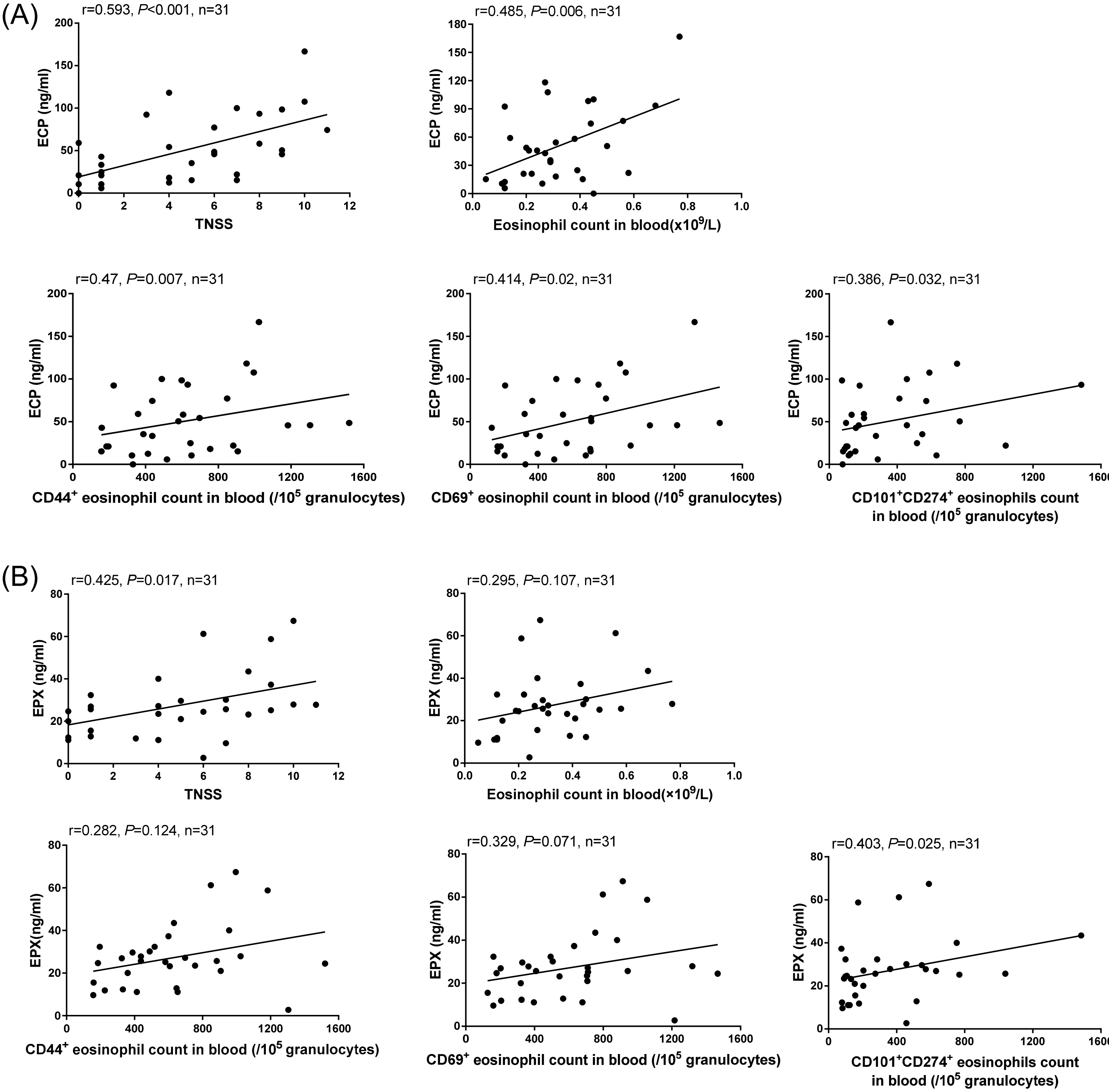
(A)
r=0.593, P<0.001, n=31
ECP (ng/ml)
TNSS
r=0.485, P=0.006, n=31
Eosinophil count in blood(x10^9/L)
r=0.47, P=0.007, n=31
CD44+ eosinophil count in blood (/10^5 granulocytes)
r=0.414, P=0.02, n=31
CD69+ eosinophil count in blood (/10^5 granulocytes)
r=0.386, P=0.032, n=31
CD101+CD274+ eosinophils count in blood (/10^5 granulocytes)
(B)
r=0.425, P=0.017, n=31
EPX (ng/ml)
TNSS
r=0.295, P=0.107, n=31
Eosinophil count in blood(×10^9/L)
r=0.282, P=0.124, n=31
EPX(ng/ml)
CD44+ eosinophil count in blood (/10^5 granulocytes)
r=0.329, P=0.071, n=31
CD69+ eosinophil count in blood (/10^5 granulocytes)
r=0.403, P=0.025, n=31
CD101+CD274+ eosinophils count in blood (/10^5 granulocytes)

Figure S4

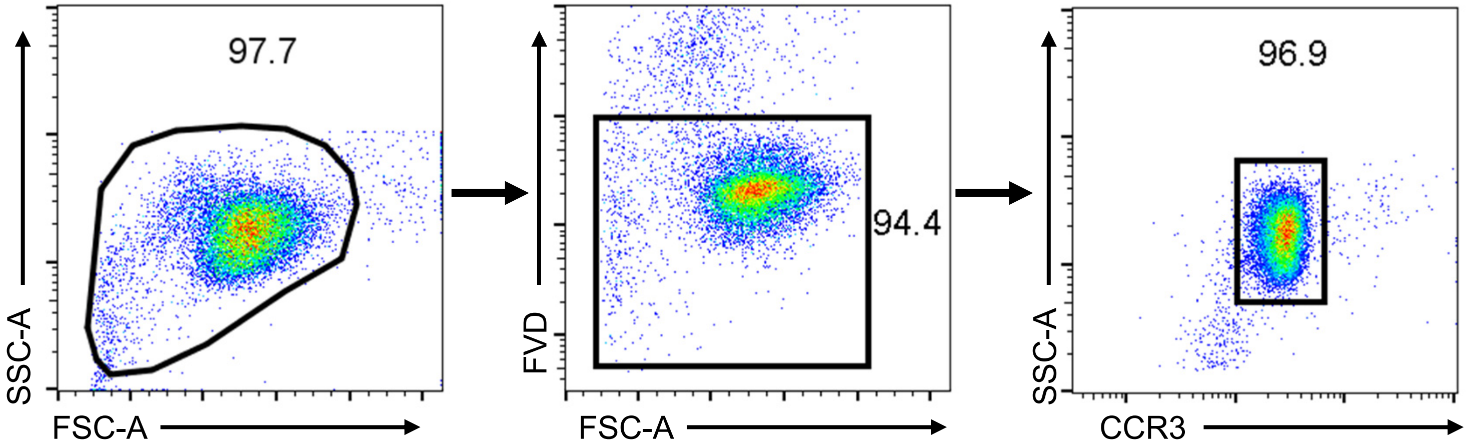

Figure S5
